# BEING RESPECTED AND ADMIRED IN OLD AGE: DYNAMICS OF SOCIAL STATUS AND AGING

**DOI:** 10.1093/geroni/igz038.2669

**Published:** 2019-11-08

**Authors:** David Weiss, Jennifer A Bellingtier, Manfred Diehl

**Affiliations:** 1 Leipzig University, Leipzig, Germany; 2 Friedrich Schiller University Jena, Jena, Germany; 3 Colorado State University, Fort Collins, Colorado, United States

## Abstract

Social status - the standing of a person or group in the social hierarchy - is part and parcel of social life and a significant determinant of cognition and behavior. Status hierarchies are basis of virtually all human and primate societies and assign different roles and privileges to its members. However, the dynamic nature of social status and the underlying mechanisms in old age are not well understood. Therefore, this symposium addresses questions of how social status is influenced by aging-related changes in roles, life events, self-concept, and images of aging and how social status shapes in response personality, subjective age, prosocial behavior, performance, and well-being in old age. Bellingtier and colleagues examined objective and subjective social status and their connections to subjective age, attitudes towards aging, and awareness of age-related changes. Zhang shows paradoxical association between aging stereotypes and prosocial behaviors toward older adults. Barber and Hamel investigated how stereotypes of reduced physical competence in old age affect the gait performance on easy and difficult tasks. Weiss and colleagues take a cross-cultural perspective on the different sources of social status in China, Germany, and the US showing that generations in contrast to age groups represent a source of high social status in later life. Finally, Kornadt investigates the dynamic interplay of changes in social roles and personality in old age. Together, these presentations enlarge our understanding of the dynamics of social status in old age.

